# Comparative transcriptome analysis of *R3a* and* Avr3a*-mediated defense responses in transgenic tomato

**DOI:** 10.7717/peerj.11965

**Published:** 2021-08-09

**Authors:** Dongqi Xue, Han Liu, Dong Wang, Yanna Gao, Zhiqi Jia

**Affiliations:** 1 College of Horticulture, Henan Agricultural University, Zhengzhou, Henan, China; 2 Henan Key Laboratory of Fruit and Cucurbit Biology, Henan Agricultural University, Zhengzhou, Henan, China

**Keywords:** Late blight, *R3a*, Plant defense, RNA-seq, Hypersensitive response

## Abstract

Late blight caused by* Phytophthora infestans* is one of the most devastating diseases in potatoes and tomatoes. At present, several late blight resistance genes have been mapped and cloned. To better understand the transcriptome changes during the incompatible interaction process between *R3a* and *Avr3a*, in this study, after spraying DEX, the leaves of MM-*R3a*-*Avr3a* and MM-*Avr3a* transgenic plants at different time points were used for comparative transcriptome analysis. A total of 7,324 repeated DEGs were detected in MM-*R3a*-*Avr3a* plants at 2-h and 6-h, and 729 genes were differentially expressed at 6-h compared with 2-h. Only 1,319 repeated DEGs were found in MM-*Avr3a* at 2-h and 6-h, of which 330 genes have the same expression pattern. Based on GO, KEGG and WCGNA analysis of DEGs, the phenylpropanoid biosynthesis, plant-pathogen interaction, and plant hormone signal transduction pathways were significantly up-regulated. Parts of the down-regulated DEGs were enriched in carbon metabolism and the photosynthesis process. Among these DEGs, most of the transcription factors, such as *WRKY*, *MYB*, and *NAC*, related to disease resistance or endogenous hormones SA and ET pathways, as well as *PR*, *CML*, *SGT1* gene were also significantly induced. Our results provide transcriptome-wide insights into *R3a* and *Avr3a*-mediated incompatibility interaction.

## Introduction

Late blight is the most important factor limiting potato production, resulting in lower yield, reduced quality, and tuber rot during storage. *P. infestans* can infect all the above-ground tissues of tomato and potato plants, as well as tubers of potatoes, especially in the open field and non-heated greenhouses under favorable conditions (10–25 °C, relative humidity >75%) (Fry, 2008). *P. infestans* has two effective life cycles: asexual and sexual. Its asexual spores (zoospores) and sporangia cannot survive for a long time in soil or dead plant debris. However, when the mating types A1 and A2 co-exist, the sexual recombination leads to the production of oospores that can survive for many years in the soil (Fernandez-Pavia et al., 2004). Recombination and rapid mutation in sexual reproduction enable the emergence of new strains and make them increasingly aggressive (Drentha & Govers, 1995; Gotoh et al., 2005). New virulent strains often increase the difficulties and challenges in potato and tomato production (Harutyunyan et al., 2008).

The safest, most effective, and environmentally friendly strategy to prevent *P. infestans* from damaging tomatoes and potatoes is to incorporate late blight resistance genes into cultivars. Resistance genes against *P. infestans* (*Rpi* genes) are mostly NB-LRR-like *R* genes, which activate the defense response by recognizing the RXLR effector secreted by *P. infestans*, leading to the hypersensitive response (HR) of cells at the infection site, limiting the expansion and colonization of germs; this process is called effector triggered immunity (ETI) (Jones & Dangl, 2006). Five race-specific tomato *Rpi* genes, *Ph-1*, *Ph-2*, *Ph-3*, *Ph-4*, and *Ph-5,* have been cloned from the wild species *S. pimpinellifolium* (Arafa et al., 2017). The mapped or cloned potato *Rpi* genes mainly include the *R1*, *R2*, *R3a*, *R3b*, *RD* and *R8* genes from *S. demissum*; *Rpi-abpt*, *Rpi-blb1*, *Rpi-blb2*, *Rpi-blb3* and *Rpi-bt1* from *S. bulbocastanum*; *Rpi-edn1.1*, *Rpi-edn1.2*, *Rpi-edn2* and *Rpi-edn3* from *S. edinense*; *Rpi-hjt1.1*, *Rpi-hjt1.2*, and *Rpi-hjt1.3* from *S. hjertingii*; *Rpi-snk1.1* and *Rpi-snk1.2* from *S. schenckii*; *Rpi-sto1*, *Rpi-sto2*, *Rpi-pta1*, *Rpi-pta2* and *Rpi-plt1* from *S. stoloniferum*; *Rpi-amr1* and *Rpi-amr3* from *S. americanum*; *Rpi-vnt1.1*, *Rpi-vnt1.2*, *Rpi-vnt1.3*, and *Rpi-vnt2* from *S. venturii*; and *R2*-like gene with unknown original species (Ballvora et al., 2010; Huang et al., 2005; Jiang et al., 2018; Kun et al., 2014; Li et al., 2011; Lokossou et al., 2009; Lokossou et al., 2010; Oosumi et al., 2009; Rodewald & Trognitz, 2013; Song et al., 2003; Wang et al., 2008; Witek et al., 2016; Witek et al., 2021).

The *R3a* gene used in this study is a well characterized *Rpi* gene, located at the major late blight resistance locus on the short arm of *S. demissum* chromosome XI (Huang et al., 2005). The *R3a* locus is highly expanded in *S. demissum* and harbors 30 to 45 *R3a* homologs per haplotype (Friedman & Baker, 2007). The *R3a* gene belongs to the CC-NB-LRR class *Rpi* gene, containing a single exon and encoding 1,283 aa. The *R3a* gene homolog (*R3aGH*) *Rpi-sto2*, cloned from *Solanum stoloniferum*, shares the same *P. infestans* strain resistance as *R3a*. *R3a* has been widely used in breeding, but many *P. infestans* strains overcome *R3a* in potato growing regions (Van Raaij et al., 2007; Rivera-Peña, 1990). The reason for this phenomenon is that the RXLR effector secreted by *P. infestans* can escape the recognition of *Rpi* through presence/absence variation (PAV), insertion/deletion (InDel), single nucleotide polymorphism (SNPs), and gene silencing (Raffaele et al., 2010; Vleeshouwers & Oliver, 2014). In *P. infestans,* two alleles of Avr3a encode secreted proteins Avr3a^*K*80∕*I*103^ (Avr3a^*KI*^) and Avr3a^*E*80∕*M*103^ (Avr3a^*EM*^). *PiAvr3a*^*KI*^ effectors were recognized by *R3a*. With the use of *R3a* varieties, *Avr3a*^*EM*^, which could evade the recognition of *R3a* and does not trigger HR, completely replaced *Avr3a*^*KI*^ and became the dominant genotype in the population (Yoshida et al., 2013). The *Avr2* fragment of the genome was deleted by the virulent strain, successfully evading the recognition of *R2* (Gilroy et al., 2011a), and the change in the stop codon position in the *Avr4* gene makes the *R4* gene ineffective (Poppel et al., 2008). The expression of *Avrblb1* does not trigger the disease resistance of *RB* plants, because the virulent strains express *ipiO4*, which is homologous to *Avrblb1*, the active combination of ipiO4 and RB that prevents RB from recognizing Avrblb1 (Chen et al., 2012; Halterman et al., 2010). *Avrblb2*, *Avr3b*, *Avrvnt1*, *AvrSmira1*, and *AvrSmira2* also have virulence alleles that can successfully escape the recognition of *Rpi* genes (Vleeshouwers & Oliver, 2014).

The recognition and resistance response of plants to late blight is a complicated dynamic process, which mainly includes two levels: pathogen-associated molecular patterns triggered immunity (PTI) and ETI. In the *Rpi*-mediated ETI response of *P. infestans*, 11 *Avr* genes of *P. infestans* have been cloned (Elnahal et al., 2020). *Rpi* genes directly or indirectly recognize the *Avr* effectors and activate the transmission of immune signals. For instance, Avr2 combines with StBSL1 to form a complex, which is specifically recognized by Rpi-R2 (Saunders et al., 2012). Avr3a binds and modifies the E3 ubiquitin ligase CMPG1, exerts a toxic function, and prevents cell necrosis caused by INF1 (Bos et al., 2010); it also targets the receptor-mediated endocytosis dynamin-related protein 2B (DRP2B) and clathrin-mediated endocytosis (CME) (Chaparro-Garcia et al., 2015). Silencing CMPG1 did not affect *Rpi-R3a’s* recognition of Avr3a and the HR response, but Co-IP confirmed that they did not interact directly (Gilroy et al., 2011b). Rpi-blb2 relies on SGT1 to recognize AVR-blb2 to activate HR response (Oh et al., 2009). In addition, the toxic RXLR effector of *P. infestans* can also infect host plants and combine target proteins to reduce plant resistance. Effector Pi03192 bound the NAC transcription factors StNTP1 and StNTP2, blocking them from entering the nucleus from the endoplasmic reticulum (Boevink et al., 2016). The combination of Pi04089 and StKRBP1 promoted the accumulation of StKRBP1 protein, promoting the infection of *P. infestans* (Wang et al., 2015). In PTI and ETI responses, the downstream signal transduction is largely overlapping; it includes reactive oxygen species burst, Ca^2+^ signaling, the MAPK pathway, and plant hormones, suggesting that PTI and ETI may share a common signaling network differently (Tsuda & Katagiri, 2010). However, compared with that during PTI, the immune signal during ETI has the characteristics of high intensity and long duration (Cui, Tsuda & Parker, 2015).

Late blight has a high evolutionary potential, and the new *Rpi* genes were quickly overcome by new *P. infestans* physiological races. Therefore, it is particularly important to explore the resistance mechanism of the *Rpi* genes and understand the signal transduction pathway of *P. infestans* resistance. The discovery of key genes in the signaling pathway will provide new ideas for the prevention of late blight. However, the potato is an autotetraploid, and the genome is highly heterozygous, which severely limits further research of the *Rpi* gene. Tomato is a model plant in pathological studies, and its genome is highly conserved. Therefore, tomato can be used to study potato *Rpi* resistance genes. In this study, to discover the changes in gene transcription levels involved in the interaction of *R3a* and *Avr3a*, we constructed transgenic plants of MM-*R3a*-*Avr3a* and MM-*Avr3a*, analyzed the differentially expressed genes (DEGs) in the metabolic pathway of disease resistance, explored the mechanisms of disease resistance. The results lay the foundation for further understanding the resistance regulatory network of *Rpi* genes*.* Additionally, it also provides plant resources for the application of the potato *Rpi* gene in the control of tomato late blight.

## Material and Methods

### Plant material and treatments

The transgenic tomato lines MM-*R3a*-*Avr3a*, MM-*Avr3a*, and *S.lycopersicum* L. cv. Moneymaker (MM) were provided by Professor Jia (Henan Agricultural University, China). For detailed information and the construction method of transgenic plants, please refer to the article published by Professor Jia in 2010 (Jia et al., 2010). *Avr3a* gene expression in transgenic plants was induced by glucocorticoid dexamethasone (DEX; Sigma, St.Louis, MO, USA). Transgenic and wild-type tomatoes were grown in a climate chamber at 21 °C with 16-h light and 8-h darkness with an ambient humidity of 95%. At the four-five leaf stage, a 0.03 mM DEX aqueous solution, containing 0.01% (w/v) Tween 20, was sprayed on the abaxial side of tomato leaves. Leaf samples were collected at 0-h, 2-h and 6-h, and three bio-replicates were employed for RNA-seq and qRT-PCR.

### Plant phenotype, Relative electric conductivity (REC), and Chlorophyll fluorescence imaging analysis

The MM- *R3a*-*Avr3a* and MM-*Avr3a* plants at the 0-h, 2-h, 4-h, 6-h, 8-h, and 12-h time points after spraying DEX were used for phenotypic observation, and the chlorophyll fluorescence content was quantified by FluorCam 800MF (Photon Systems Instruments, spol. s ro District Brno-City, Czech Republic). The maximum quantum yield of photosystem II photochemistry (Fv/Fm) can display a strong contrast between infected and healthy tissues (Rousseau et al., 2013). We measured the Fv/Fm values of tomato transgenic plants according to Murchie’s method to quantify the severity of the HR (Murchie & Lawson, 2013). One-way analysis of variance (ANOVA) was performed by SPSS 17.0 (SPSS Corp., Chicago, USA) and followed up with a least-significant difference post hoc test (*α* = 0.05). REC is the major indicator of membrane damage, and the REC of the tomato leaf was calculated according to Cottee’s protocol (Cottee et al., 2007).

### RNA extraction, library construction, and sequencing

Total RNA from foliage samples of MM, MM- *R3a-Avr3a*, and MM-*Avr3a* plants after spraying DEX, with three bio-replicates, were extracted as described in the literature (Yang et al., 2020). High integrity RNA was use to construct the sequencing library. Libraries were generated using NEB Next^®^ Ultra™ RNA Library Prep Kit for Illumina^®^ (NEB, USA). The library quality was assessed using the Agilent Bioanalyzer 2100 system and RNA-sequencing was performed using the Illumina Hiseq2500 platform hosted by Biomarker Technologies CO., LTD (BTC, Beijing, China; http://www.biomarker.com.cn/); and 150-bp paired-end reads were generated.

### Quality control, mapping, and functional annotation

Raw reads were first processed using in-house Perl scripts developed by BTC. Next, clean reads were obtained by removing adapter fragments, ploy-N and low quality reads, and Q20, Q30, and GC content were calculated. Clean reads were mapped to the tomato reference genome database (https://www.ncbi.nlm.nih.gov/genome/7?genome_assembly_id=393272) by using HISAT2 software (https://daehwankimlab.github.io/hisat2/). Gene function was annotated based on Nr, Nt, Pfam, KOG/COG, and the Swiss-Port bioinformatics database according to Zhu’s method (Zhu et al., 2014).

### Quantification and analysis of DEGs

The quantification of gene expression levels was estimated by fragments per kilobase of transcript per million fragments mapped (FPKM) and normalized using HTseq v0.9.1 (Anders, Pyl & Huber, 2015; Trapnell et al., 2010). Differential expression analysis of two samples was performed using the DESeq2 package (Love, Huber & Anders, 2014). The *P* values were adjusted by using Benjamini–Hochberg’s method to control the false discovery rate (FDR) (Benjamini & Hochberg, 1995), the genes with a fold change ≥2 or ≤-2 and an adjusted *P* value (*padj*) <0.01 were designated as DEGs.

### GO and KEGG enrichment analysis

GO enrichment analysis of DEGs was conducted by using the GO-seq R package. This analysis was based on Wallenius noncentral hypergeometric distribution (Young et al., 2010), and the GO terms with an adjusted *P* value and *FDR* <0.01 were considered as indicating significant enrichment. KEGG Orthology Based Annotation System (KOBAS) 3.0 software was used to analyze the significant enrichment KEGG pathways of DEGs (Xie et al., 2011).

### Weighted gene Co-expression network analysis (WGCNA)

WGCNA can be used to analyze the expression patterns of genes between multiple samples, cluster genes with similar expression patterns, and analyze the correlation between modules and specific traits or phenotypes. Therefore, we used WGCNA to identify the specific modules of co-expressed genes associated with incompatible interactions between *R3a* and *Avr3a* genes. WGCNA was performed according to Langfelder and Horvath (Langfelder & Horvath, 2008). WGCNA adopts the dynamic hybrid tree cut algorithm. Parameters settings are FPKM >= 1, minimum module size is 30, and Module similarity threshold is 0.25. For modules screened by WGCNA, module eigengene was calculated *via* PCA, GO, and KEGG pathway enrichment was performed to analyze the biological functions of DEGs in the modules.

### Real-time quantitative PCR (qRT-PCR) analysis

Twenty DEGs involved in plant-pathogen interaction and plant hormone signal transduction pathways were selected for qRT-PCR, and primers were designed using Premier 5 (Table S1). qRT-PCR was performed using ChamQ Universal SYBR qPCR Master Mix (Vazyme, Nanjing, China) on a CFX96™ Real-Time System (Bio-Rad, USA) with the cycle steps of pre-degeneration at 95 °C, 3min, and 1 cycle; followed by 40 cycles of 95 °C for 10s, and 60 °C for 40s; and melting curve analysis at 95 °C for 10s, 65 °C for 5s, and 95 °C for 5s. The relative expression level of each gene was calculated by the 2^−ΔΔCt^ method (Livak & Schmittgen, 2001), and the correlation coefficients between RNA-seq data and qRT-PCR were evaluated using GraphPad Prism 9 (San Diego, CA, USA).

## Results

### Symptoms of MM-*R3a-Avr3a* and MM-*Avr3a* after DEX treatment

**Figure 1:**
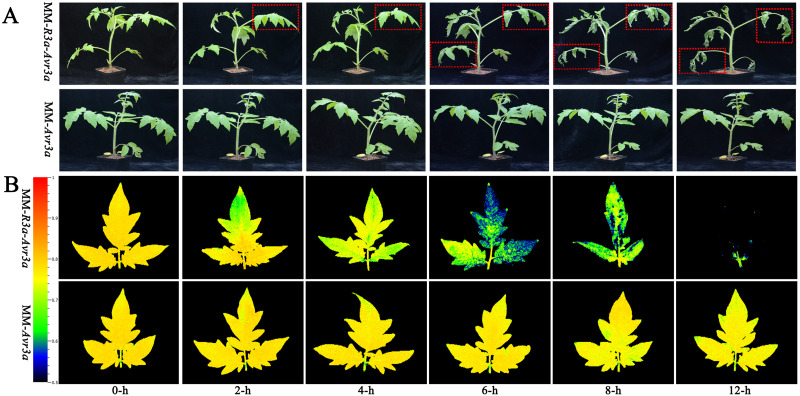
Phenotypic symptoms of MM-R3a-Avr3a and MM-Avr3a plants at different time points after spraying DEX.

The DEX induced *Avr3a* expression in transgenic MM-*R3a*-*Avr3a* tomatoes, and Avr3a can trigger R3a-mediated HR. To identify the different stages of the development of HR symptoms, we investigated the phenotype and chlorophyll fluorescence of the transgenic plants after spraying DEX. The phenotypic observation revealed no significant difference in MM-*R3a*-*Avr3a* tomato between 2-h and 4-h, but the leaves began to wilt at 6-h. The symptoms of wilting of the whole plant were obvious at 8-h, and all leaves including the growth point were severely crinkled at 12-h and the petiole become soft. A stronger and more rapid systemic HR was induced in the MM-*R3a*-*Avr3a* line, whereas no symptoms were observed in the MM-*Avr3a* line (Fig. 1A).

**Figure 2:**
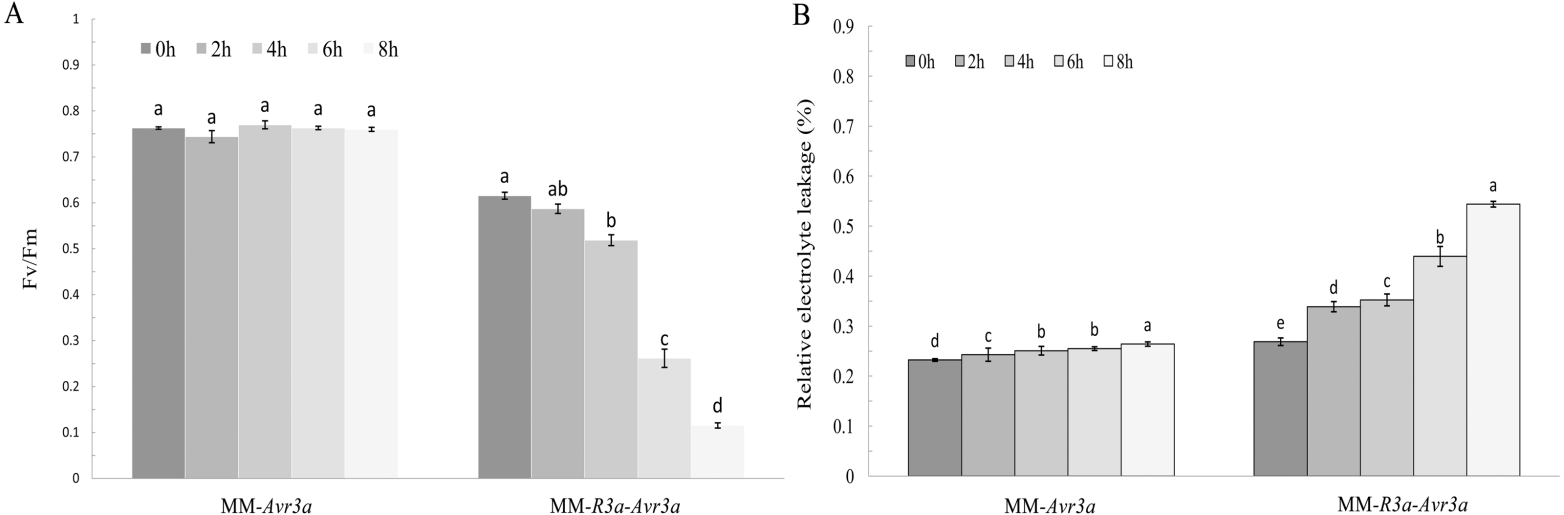
Significance analysis of FvFm and REC at different time points.

Notably, the chlorophyll fluorescence imaging directly shows the region where the symptoms of leaf wilting occur. MM- *Avr3a* plants had a higher level of Fv/Fm than MM-*R3a*-*Avr3a* plants, and no difference in Fv/Fm values was observed in MM-*Avr3a* plants from 0 h to 8 h (Fig. 1B). For MM-*R3a-Avr3a* plants, the Fv/Fm value showed a significant downward trend after spraying of DEX; no significant difference was observed between 2-h and 0-h. However, a significant difference between 4-h and 0-h, and an extremely significant difference between 6-h, 8-h, and other time points (*P* < 0.01, Fig. 2A) were observed. The REC values of MM-*Avr3a* plants at different time points were low, but they significantly differed from each other. However, MM-*R3a*-*Avr3a* plants showed an obvious increase in REC after spraying DEX, indicating impairment of growth processes (Fig. 2B). The results of REC were consistent with those of chlorophyll fluorescence.

### Transcriptome sequencing and mapping

According to the preliminary identification results of plant phenotypes, the tomato leaves of MM- *R3a*-*Avr3a* and MM-*Avr3a* at 0-h, 2-h, and 6-h after spraying DEX were used for transcriptome sequencing to parse the reaction process of *R3a* and *Avr3a* incompatible interactions. In addition, 0-h MM was sequenced and compared with the transcripts of 0-h MM-*R3a*-*Avr3a* and 0-h MM-*Avr3a* 0 h, to analyze the transcriptional differences caused by the transfer of *R3a* and *Avr3a* genes. After sequencing quality control, 145.26 Gb clean reads were obtained. The percentage of Q30 bases in each sample was more than 93.56%, and the average GC content was 43.24%. The alignment result with the reference genome shows that 94.68%–97.67% clean reads per sample aligned with the reference genome, and 91.29%–94.18% reads were uniquely mapped (Table S2). Additionally, the Pearson correlation coefficient (*R*^2^) of different biological replicates was between 0.9436 and 0.9818, revealing a high level of reproducibility of RNA expression patterns (Fig. S1).

### Identification and annotation of DEGs

**Figure 3:**
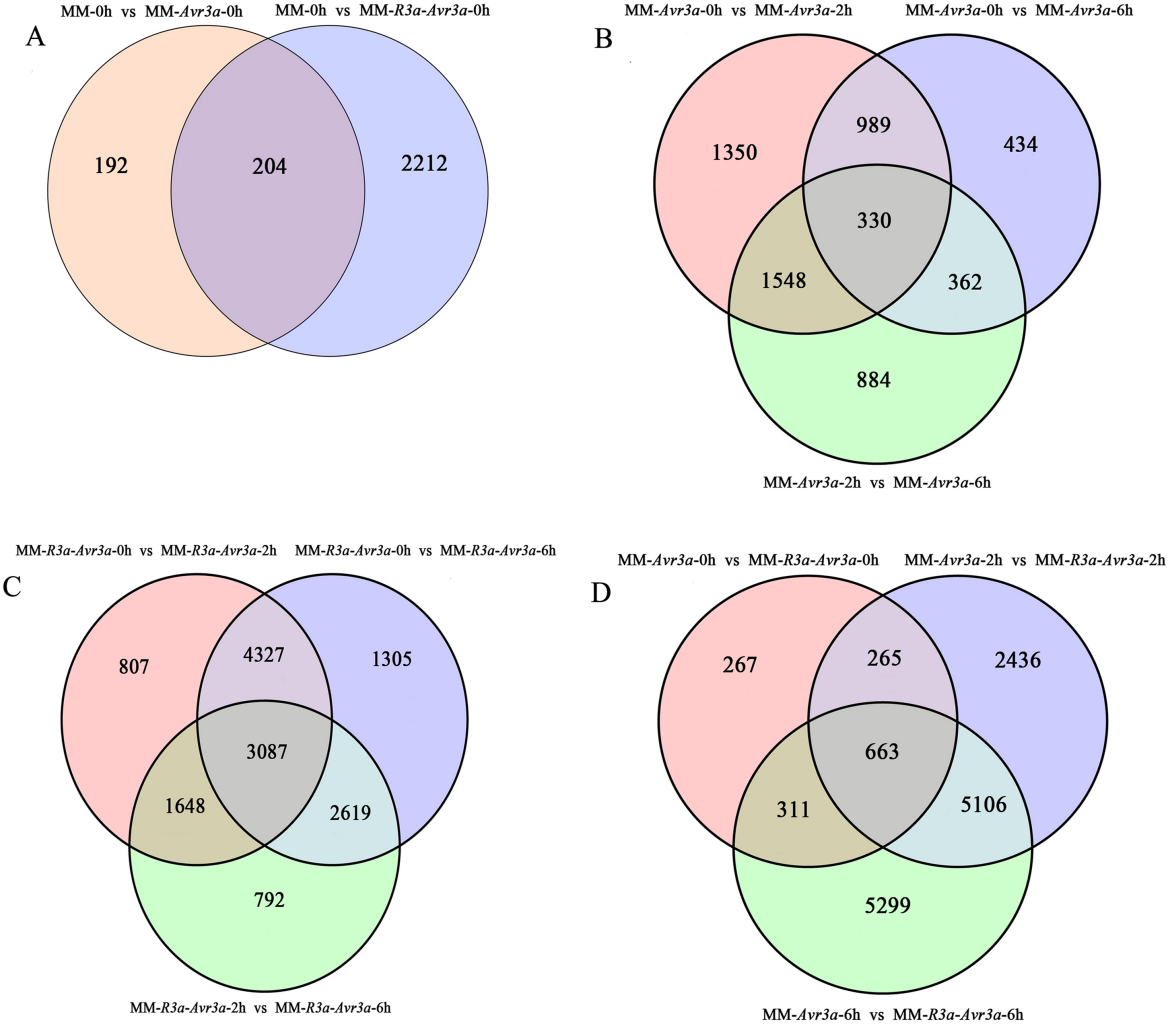
Venn diagram of the relationship between differentially expressed gene groups.

A total of 16,336 DEGs were detected, and the FPKM values of gene expression are listed in Table S3. A total of 396 DEGs were detected in MM-*Avr3a* vs MM, and 2,416 DEGs were enriched in MM-*R3a*-*Avr3a* vs MM (Table S4, Fig. 3A). Compared with MM-*Avr3a* 0-h, the total DEGs decreased from 4,217 to 3,124 at 2-h and 4-h, especially down-regulated genes greatly reduced. We detected 1,319 overlapping DEGs were at 2-h and 6-h compared with 0-h in MM-*Avr3a*, of which 330 genes showed the same expression pattern at 2-h and 6-h (Table S4, Fig. 3B). In MM-*R3a*-*Avr3a* plants, the DEGs at 2-h were slightly more reduced than those at 6-h, but the total DEGs were much higher than those in MM-*Avr3a*. A total of 7,324 repeated DEGs were detected in MMR3a-Avr3a plants at 2-h and 6-h, and 729 genes were differentially expressed at 6-h compared to those at 2-h (Table S4, Fig. 3C). The statistical results of DEGs at the same time point between MM-*R3a*-*Avr3a* and MM-*Avr3a* showed that 1,506, 8,470, and 11,379 DEGs were detected at 0-h, 2-h, and 6-h, respectively, showing a significant and rapid increasing trend. Notably, 5,106 genes were specifically expressed at 2-h and 6-h in MM-*R3a*-*Avr3a* tomatoes (Table S4, Fig. 3D). Functional annotation of DEGs was conducted, and the number of genes annotated for each differentially expressed gene set is shown in Table S5.

### GO and KEGG pathways functional enrichment analysis of DEGs

The enrichment of GO and KEGG pathways of DEGs can help us understand in detail the mechanism that R3a specifically recognizes Avr3a and triggers HR response. For MM-*R3a*-*Avr3a* tomatoes, we observed the significantly enriched jasmonic acid biosynthetic process (JA) and regulation of the JA-mediated signaling pathway, photosynthesis, thylakoid membrane organization and intracellular signal transduction biological process at 2-h and 6-h. In the molecular function category, oxidoreductase activity, phenylalanine ammonia-lyase activity, and calcium ion binding were all enriched (Figs. S2A and S2B). This finding is consistent with the pathways in which DEGs were significantly enriched in the KEGG (Figs. S2C and S2D). For the MM-*Avr3a* tomato, at 2-h and 6-h, we observed significantly enhanced oxidation–reduction and photosynthesis, and the molecular function of oxidoreductase activity (Figs. S3A and S3B). Additionally, the predominant pathways identified were those of carbon metabolism (78 genes) and photosynthesis (29 genes). In addition, the plant hormone signal transduction pathway and photosynthesis were predominant at 6-h, evidenced 47 and 22 DEGs,respectively (Figs. S3C and S3D).

### Validation of RNA-seq data by real-time quantitative PCR

To validate the results of RNA-seq data, we randomly selected 20 DEGs in tomato MM- *R3a*-*Avr3a* plants at 6-h point after spraying DEX (Table S1). These genes were significantly enriched in pathways closely related to plant disease resistance, such as the JA biosynthetic process, phenylalanine metabolism, plant-pathogen interaction, and plant hormone signal transduction. The correlation analysis of expression levels of 20 DEGs in RNA-seq and qRT-PCR showed that the expression patterns of these genes were consistent and had a strong positive correlation (*R*^2^ =0.9235; Fig. S4), confirming the reliability of the RNA-seq data.

### Construction of gene Co-expression networks

**Figure 4:**
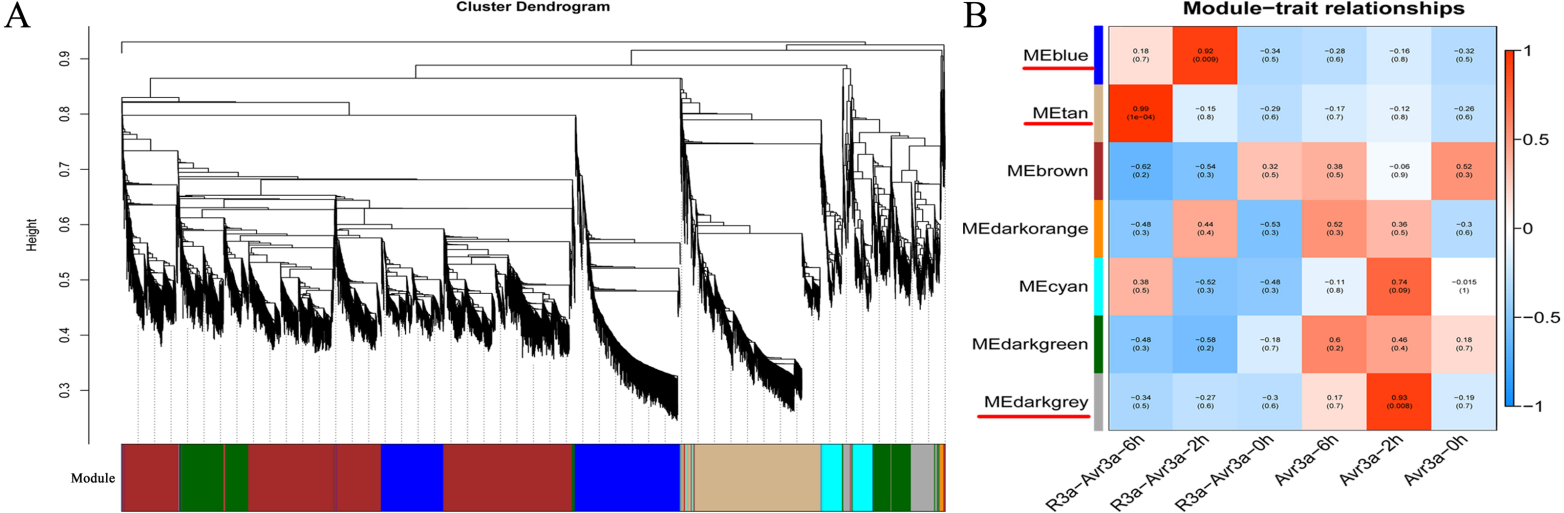
WGCNA of DEGs in MM-R3a-Avr3a and MM- Avr3a at different time point.

After filtering the low-quality DEGs (FPKM <1), 9,228 genes were generated from WGCNA analysis. The WGCNA results showed that DEGs can be subdivided into seven modules (marked with different colors; Fig. 4A). Especially, the genes in the same module have a high correlation coefficient. Three of the seven co-expression modules were selected that have the highest correlation degree with one of the samples. In Fig. 4B, the three modules are indicated with red underlines. The blue module comprised 1,743 genes specific to the MM-*R3a*-*Avr3a*_2-h group. The GO and KEGG enrichment results of genes in blue modules are shown in Table S6. The tan module, with 1,605 identified genes, was highly associated with the MM-*R3a*-*Avr3a*_6-h group, and the annotation information of genes is shown in Table S7. The dark grey module, representing 438 genes, was highly associated with MM-*Avr3a*_2-h. Details of gene annotation are shown in Table S8.

Hub gene, an important node in the gene network constructed by WGCNA, has high connectivity. We enriched 406, 435, and 80 hub genes in three modules of blue, tan, and dark grey, respectively. The KEGG pathway enrichment analysis results of the hub genes in the three modules showed that phenylpropanoid biosynthesis (ko00940), plant-pathogen interaction (ko04626), carbon metabolism (ko01200), and plant hormone signal transduction pathways (ko04075) were significantly enriched in MM- *R3a*-*Avr3a* at 2-h and 6-h. Additionally, the genes enriched in the peroxisome (ko04146) and glutathione metabolism (ko00480) pathways at 6-h were significantly higher than those at 2-h (Fig. S5). The GO analysis results of the hub gene in the blue and tan modules of the MM-*R3a*-*Avr3a* material showed that cell killing (GO: 0031640) in the biological process, metallochaperone activity (GO: 0004222), and guanyl-nucleotide exchange factor activity (GO: 0008928) in the molecular function were also significantly annotated at 6-h, but not detected at 2-h (Fig. S6). Enrichment results of the WGCNA module were consistent with the prior GO and KEGG analysis results of MM-*R3a*-*Avr3a* tomato at 2-h and 6-h. Although the aforementioned six KEGG pathways mentioned were also enriched in the dark grey module of MM-*Avr3a* 2-h tomato, the number of DEGs in the pathways was extremely small.

## Discussion

Late blight has a high evolutionary potential, and new *Rpi* genes can be quickly overcome by new *P. infestans* physiological races, which limit the utilization of *Rpi* genes. Therefore, exploring the disease-resistance mechanism of *Rpi* genes is particularly important for understanding the disease resistance signal transduction pathway. In this study, we conducted a comparative transcriptome analysis between MM-*R3a*-*Avr3a* and MM-*Avr3a* at different time points. The results provide comprehensive information on genes involved in the incompatible interaction process of *Rpi* gene *R3a* and *P. infestans Avr3a*.

According to the GO, KEGG pathway and WCGNA analysis, the JA biosynthetic process (GO: 0009695), phenylpropanoid biosynthesis (ko00940), plant-pathogen interaction (ko04626), and plant hormone signal transduction pathways (ko04075) were significantly enriched in MM- *R3a*-*Avr3a* lines. This result is similar to the transcriptome enrichment results of the incompatible interaction between *RB* gene and *P. infestans* race US940480 (Gao & Bradeen, 2016). Additionally, comparative analysis of defense responses of *R1*, *R3a*, and *R3b* transgenic potato lines with different *Rpi* genes to *P. infestans* race CN152 and 89148 indicated that defense pathways of the three *R* genes were similar. For example, plant-pathogen interaction, and pentose and glucuronate interconversions (ko00040), were specifically enriched but still had minor differences (Yang et al., 2020), and this finding was also proved in our research. Additionally, with the extension of the incompatible interaction time between R3a and Avr3a, the wilting degree of plant leaves intensified, and some down-regulated DEGs were enriched in carbon metabolism and photosynthesis process. This finding indicates that the priority of metabolism shifted from photosynthesis to pathogen defense. Similar metabolic shifts have been found in *Arabidopsis thaliana* under pathogen attack (Depuydt et al., 2009). However, for MM-*Avr3a* lines, most of the pathways associated with plant disease resistance have not been detected.

The process of co-evolution between pathogens and plants has formed a complex and effective defense mechanism. When plants are invaded by pathogens, complex defense responses are triggered. Disease resistance signals are transmitted from the infected site to the whole plant by endogenous signal molecules, causing systemic resistance in plants. GO and KEGG analysis of DEGs demonstrated that t phenylalanine metabolism process (GO: 0006559) was significantly up-regulated in MM- *R3a*-*Avr3a* lines. Phenylalanine is downstream of the shikimic acid pathway and mediates the biosynthesis of SA. Additionally, the downstream genes of SA, *NPR1* (*Solyc02g069310.3*, *Solyc07g040690.3*, *Solyc07g044980.3*, ko04075), and *PR-1* (*Solyc01g106620.2*, ko04626), were significantly up-regulated at 2-h and returned to normal levels at 6-h; however, they were not differentially expressed in MM-*Avr3a*. The transcriptome analysis results of the incompatible interactions of three different *Rpi* genes with *P. infestans* were also enriched in these DEGs genes (Yang et al., 2020). In addition, several DEGs involved in the ethylene signal transduction pathway were identified. Combined with the measurement results of the ethylene content of MM-*R3a*-*Avr3a* plants at different time points after spraying DEX (Fig. S7), we observed that the ethylene content increased rapidly at 2-h, and the expression levels of *CTR1* (*Solyc09g009090.3*, *Solyc10g083610.2*, ko04075) and ethylene receptor (*ETR*, *Solyc05g055070.4*, *Solyc06g053710.3*, *Solyc12g011330.4*, ko04075) were up-regulated. Similarly, Flg22 was found to induce ET production at 1-h that peaked at 4-h in *Arabidopsis* seedlings (Liu & Zhang, 2004). The ethylene molecule binds to ETR or ERS receptors and inactivates CTR1 without phosphorylating EIN2 (Ju et al., 2012; Qiao et al., 2012). The C-terminus of EIN2 was cut off and transported to the nucleus, where the expression levels of EIN3 (*Solyc01g006650.2*, *Solyc01g014480.3*, ko04075) and ethylene response factor (*Solyc03g005520.1*, *Solyc09g089930.3*, ko04075) were also sharply increased, regulating the rapid expression of ethylene response genes. Additionally, the JA biosynthetic process was up-regulated, and JA production was usually induced by necrotrophic pathogen infections in potato, such as those caused by *S. sclerotiorum* and *Colletotrichum coccodes* (Halim et al., 2009).

R3a protein specifically recognizes Avr3a and mediates HR response. We identified three pathogenesis-related 1 (*PR*-*1*, *Solyc01g106620.2*, *Solyc09g007010.1*, *Solanum*_*lycopersicum*_*newGene*_*3034*, GO:0031640) genes involved in HR that were up-regulated in MM-*R3a*-*Avr3a*. *PR-1*, as a marker gene of systemic acquired resistance (SAR) and downstream defense, is an important part of the plant defense gene against *P. infestans* (Faino et al., 2010; Melgar, Abney & Vierling, 2006). Additionally, incompatible interaction leads to the production of key transcription factors (TFs), which coordinate the expression of downstream target genes (Orłowska et al., 2012). WRKY TFs bind to the W-box in promoters of pathogen-responsive genes, such as *PR-1*, *PR-2*, *PR-3*, and *PR-5*, and are often co-expressed during SAR (Eulgem et al., 2000; Orłowska et al., 2012; Van Verk et al., 2008). Six highly expressed WRKY TFs have been screened: *Solyc06g066370.4*, *Solyc09g014990.4*, *Solyc01g095100.4*, *Solyc10g011910.4*, *Solyc12g006170.2*, and *Solyc07g066220.3* (ko04626). Other TFs, such as NAC (*Solyc06g061080.3.1* and *Solyc03g115850.3.1*, GO:0006355), MYB (*Solyc02g067760.3.1*, GO:0003677), and ABCG (*Solyc05g054890.3.1*), were also significantly up-regulated. Potato NAC43 and MYB8-mediated transcriptional regulation of the secondary cell wall biosynthesis inhibit *P. infestans* infection (Yogendra et al., 2017). TF *StWRKY1* regulates the metabolites of phenylpropanoid and makes potatoes resistant to late blight (Yogendra et al., 2015). Some TFs identified in this study may play an important role in the regulation of *R3a* against *P. infestans*. In addition, genes related to late blight resistance, such as *SG*T1 (*Solyc03g007670.4*, *Solyc06g036420.3*, ko04626), and cyclic nucleotide-gated cation channel (*CNGC*, *Solyc03g007260.3*, *Solyc03g098210.4*, *Solyc05g050350.2*, *Solyc05g050360.3*, *Solyc05g050380.4* and *Solyc06g051920.4*, ko04626), were also significantly up-regulated. Although *EDS1*, *RAR1* and *HSP90* were not required in *Rpi-blb2*-mediated late blight resistance, these genes were also significantly enriched in MM-*R3a*-*Avr3a* plants (Oh, Kwon & Choi, 2014).

## Conclusions

In this study, we performed GO, KEGG, and WGCNA analysis on DEGs of MM- *R3a*-*Avr3a* and MM-*Avr3a* plants at different time points. The transcriptome process of the incompatible interaction between the *R3a* gene and *Avr3a* was preliminarily analyzed. When R3a specifically recognized Avr3a, downstream defense signaling transductions were activated, for example, by significantly up-regulating the expression of *CNGC*, *RBOH* and calcium-binding protein CML (*CaMCML*), which led to the rapid transient generation of reactive oxygen species and nitric oxide (NO), as well as the rapid and drastic increase in Ca^2+^. Subsequently, the plant hormone signal transduction pathways, such as SA and ET, were rapidly activated, and defense-related TFs, such as WRKY, MYB, and NAC, were triggered, and the whole-plant HR was observed in MM-*R3a*-*Avr3a*.

##  Supplemental Information

10.7717/peerj.11965/supp-1 Supplemental Information 1 RNA-Seq correlation analysis between samples

10.7717/peerj.11965/supp-2 Supplemental Information 2 GO and KEGG pathway enrichment analysis of DEGs in MM-*R3a*-*Avr3a* tomato

10.7717/peerj.11965/supp-3 Supplemental Information 3 GO and KEGG pathway enrichment analysis of DEGs in MM-*Avr3a* tomato

10.7717/peerj.11965/supp-4 Supplemental Information 4 Correlation of expression levels between RNA-seq and qRT-PCR

10.7717/peerj.11965/supp-5 Supplemental Information 5 KEGG pathway analysis of the module hub genes

10.7717/peerj.11965/supp-6 Supplemental Information 6 Classification and statistics of GO annotations for hub genes in the blue and tan modules

10.7717/peerj.11965/supp-7 Supplemental Information 7 Ethylene content of transgenic plants at different time points

10.7717/peerj.11965/supp-8 Supplemental Information 8 The primers for qRT-PCR

10.7717/peerj.11965/supp-9 Supplemental Information 9 Basic statistical of RNA-sequencing data

10.7717/peerj.11965/supp-10 Supplemental Information 10 DEGs between MM, MM-*R3a*-*Avr3a* and MM-*Avr3a* at different time points

10.7717/peerj.11965/supp-11 Supplemental Information 11 The statistical of differentially expressed genes at each time point between different samples

10.7717/peerj.11965/supp-12 Supplemental Information 12 Functional annotation statistical results of DEGs

10.7717/peerj.11965/supp-13 Supplemental Information 13 GO and KEGG enrichment results of genes in blue modules

10.7717/peerj.11965/supp-14 Supplemental Information 14 GO and KEGG enrichment results of genes in tan modules

10.7717/peerj.11965/supp-15 Supplemental Information 15 GO and KEGG enrichment results of genes in dark grey modules

10.7717/peerj.11965/supp-16 Supplemental Information 16 Data of FvFm and REC (Fig. 2)

10.7717/peerj.11965/supp-17 Supplemental Information 17 Data of correlation analysis of 20 DEGs in RNA-seq and qRT-PCR (Fig. S4)

## Additional Information and Declarations

### Data Availability

The following information was supplied regarding data availability: The data of correlation analysis of RNA-seq and qRT-PCR, and significance analysis of Fv/Fm and REC are available in the Supplemental Files. Raw sequencing data for 21 samples are available at the NCBI SRA database: PRJNA693866; SRR13556529, SRR13556528, SRR13556527, SRR13556526, SRR13556525, SRR13556524, SRR13556523, SRR13556522, SRR13556521, SRR13556520, SRR13556519, SRR13556518, SRR13556538, SRR13556537, SRR13556536, SRR13556535, SRR13556534, SRR1355653, SRR13556532, SRR13556531, SRR13556530.
