# Supplementary figures and images for: Comparative transcriptome analysis of R3a and Avr3a-mediated defense responses in transgenic tomato
Source: PeerJ. 2021 Aug 9;9:e11965. doi: 10.7717/peerj.11965 (PMC8359799; doi:10.7717/peerj.11965)

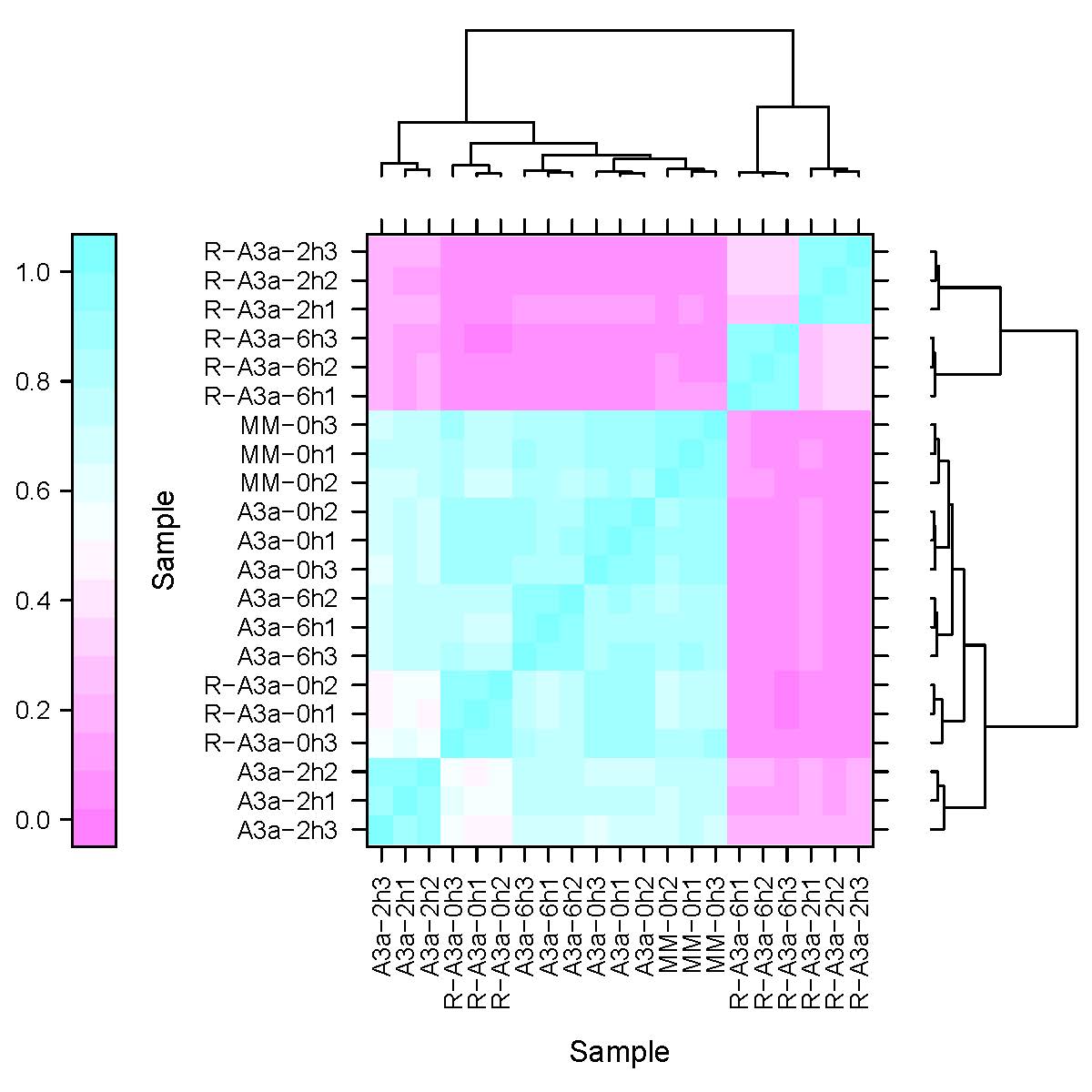

Supplement: Supplemental Information 1 [file peerj-09-11965-s001.jpg]

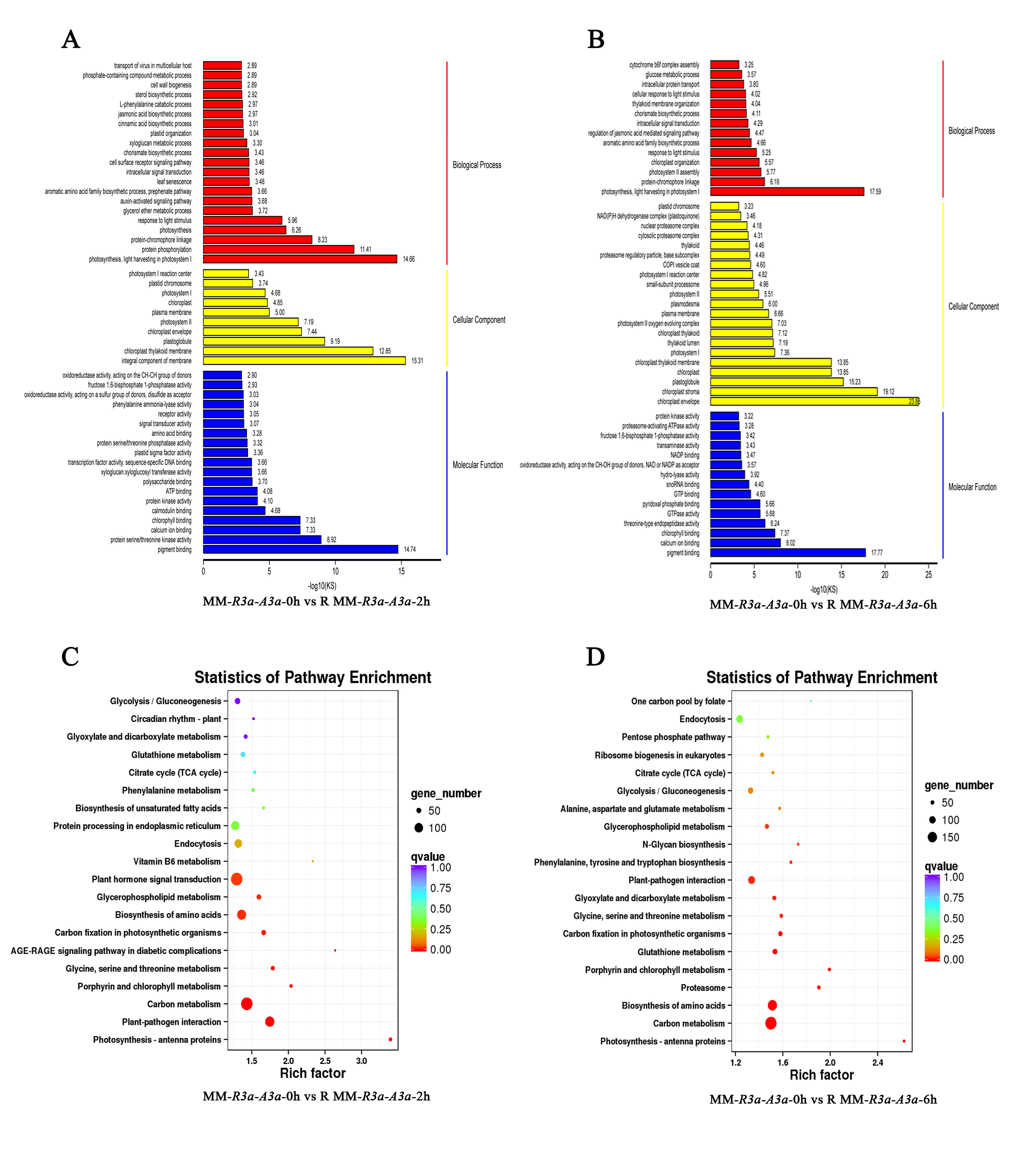

Supplement: Supplemental Information 2 [file peerj-09-11965-s002.jpg]

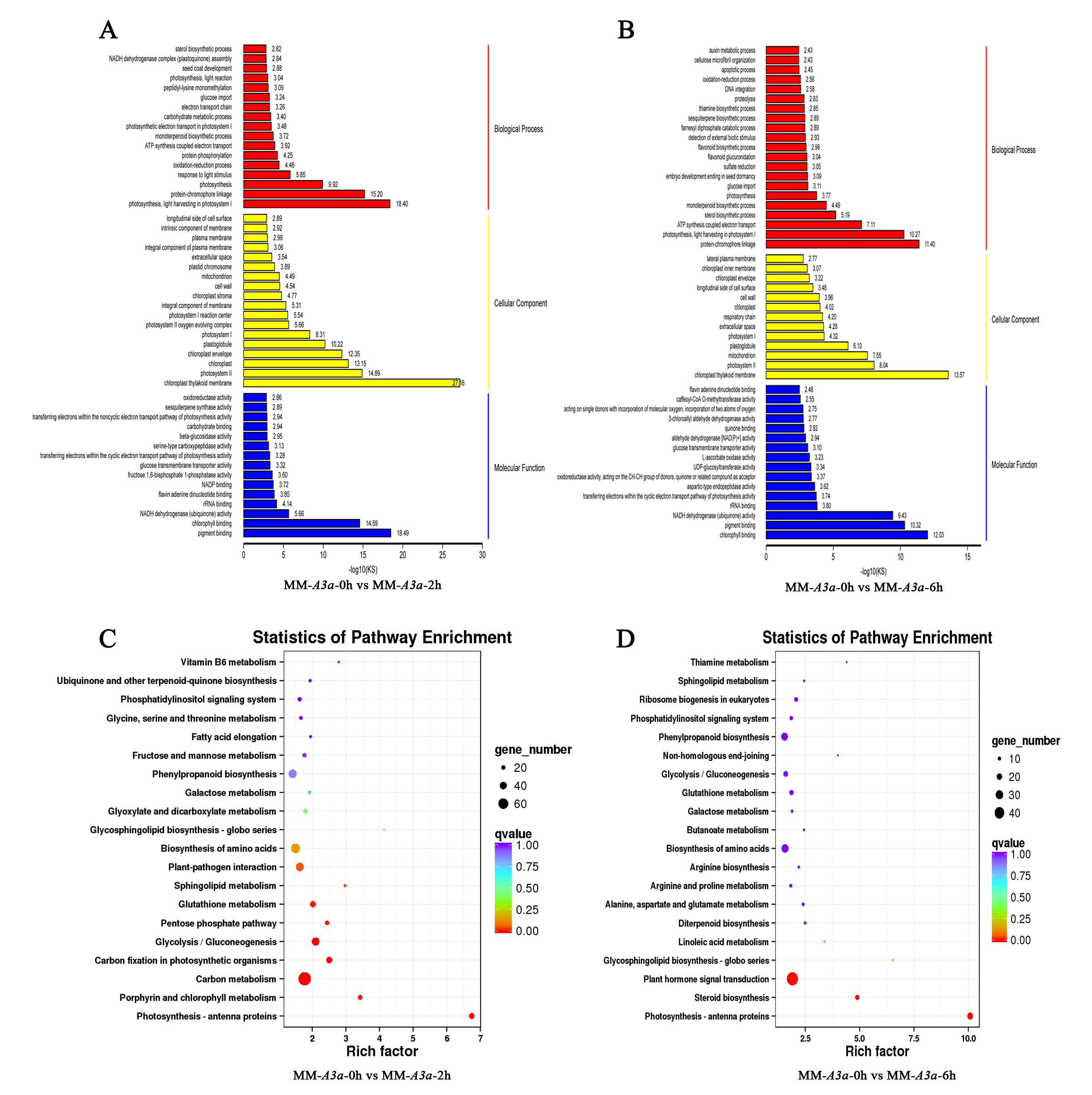

Supplement: Supplemental Information 3 [file peerj-09-11965-s003.jpg]

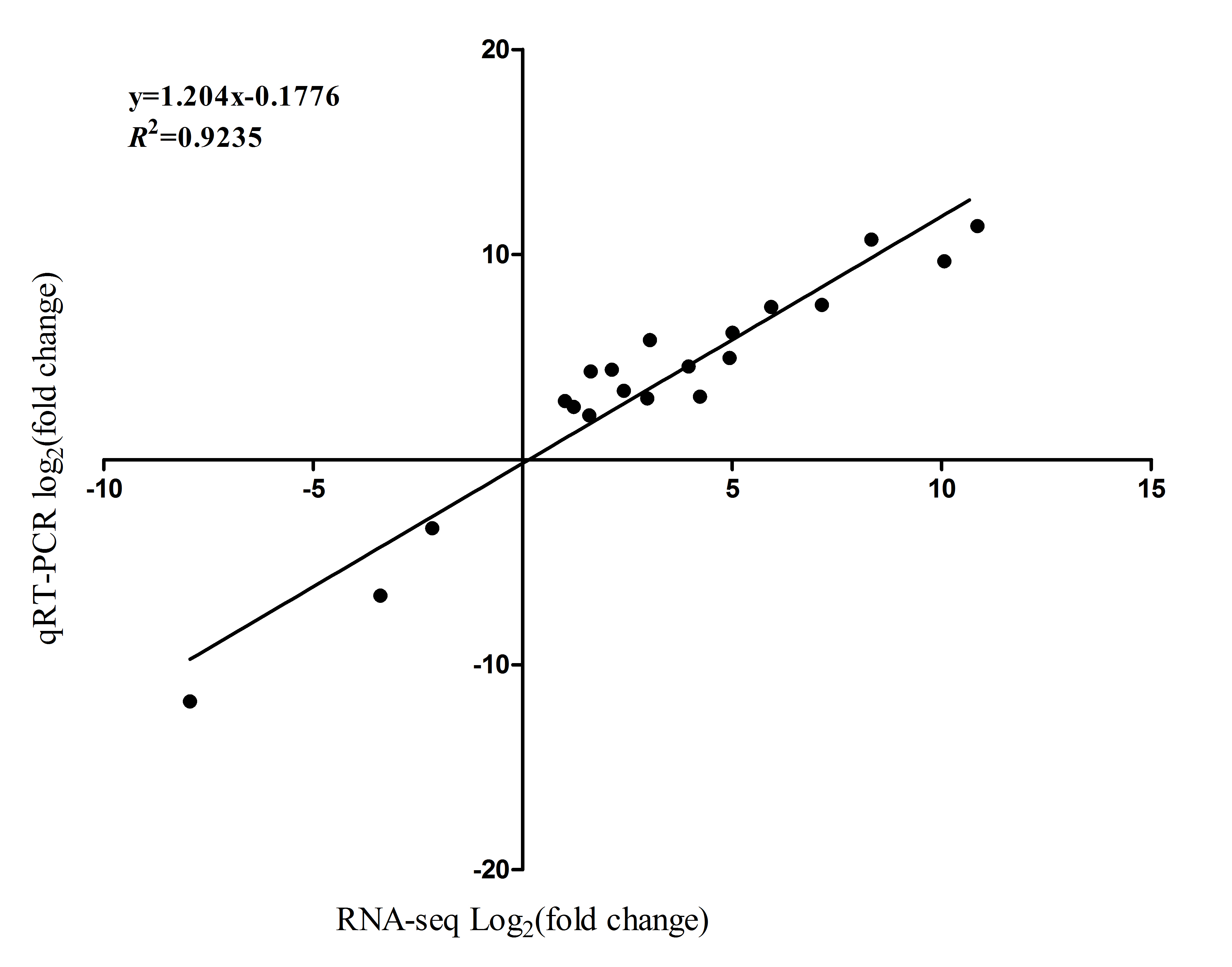

Supplement: Supplemental Information 4 [file peerj-09-11965-s004.jpg]

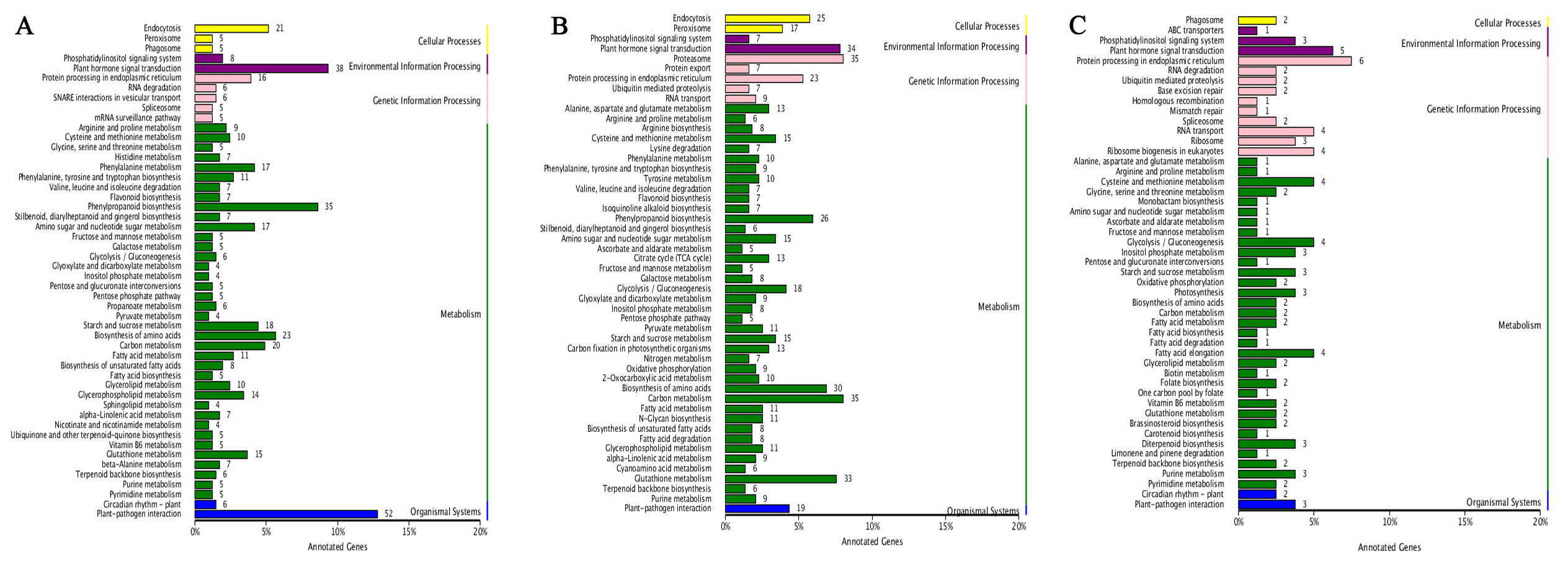

Supplement: Supplemental Information 5 [file peerj-09-11965-s005.jpg]

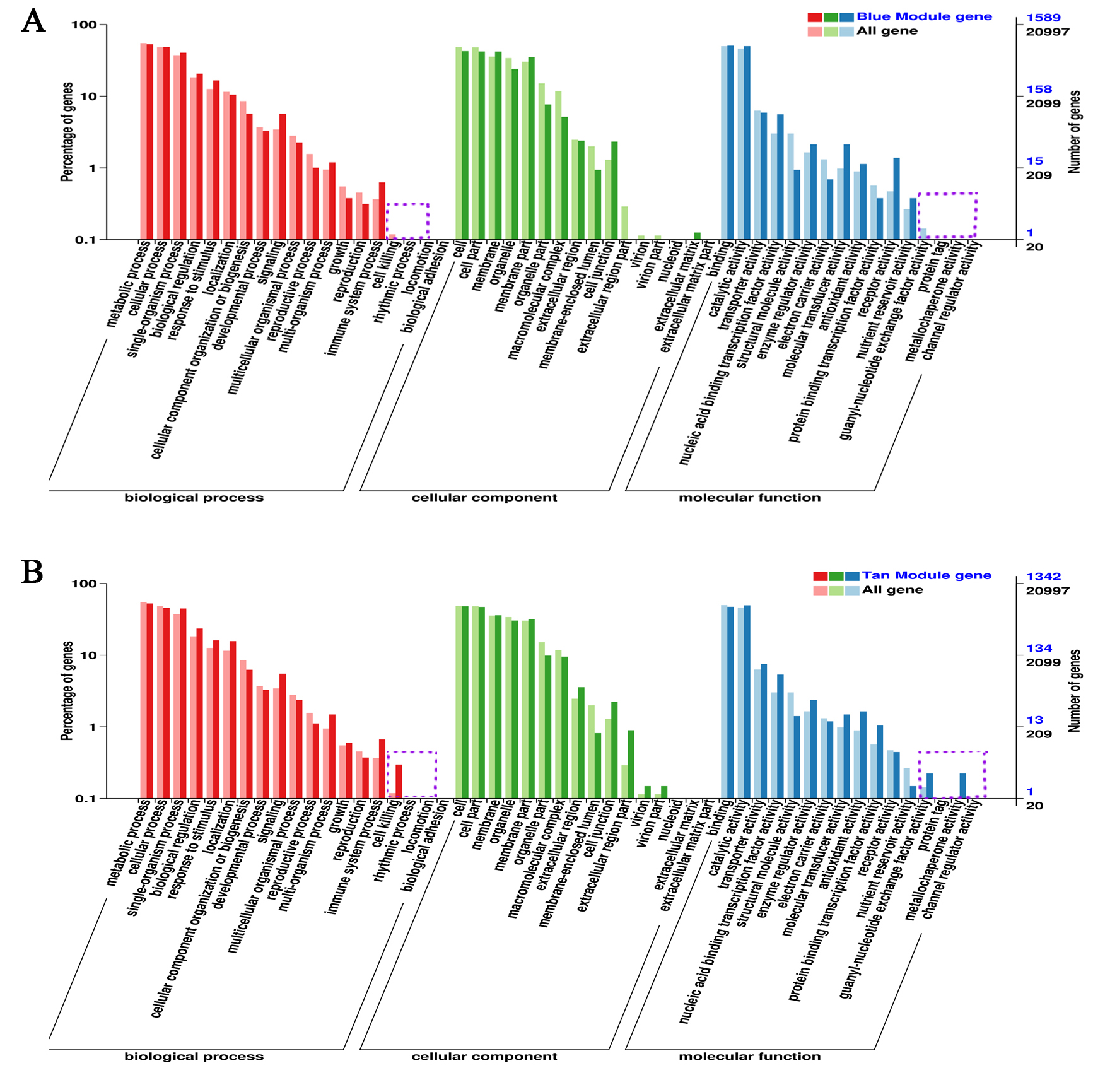

Supplement: Supplemental Information 6 [file peerj-09-11965-s006.jpg]

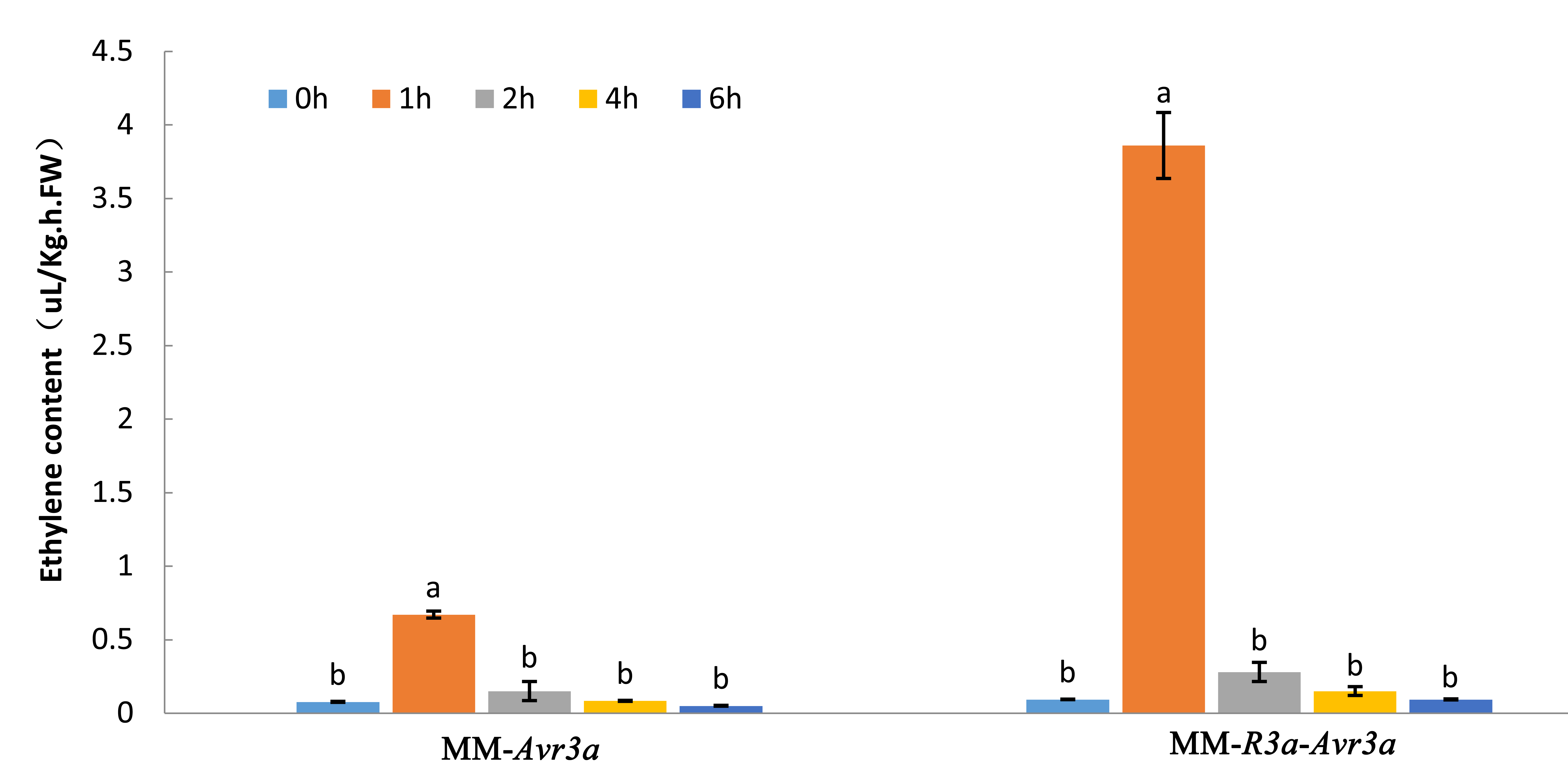

Supplement: Supplemental Information 7 [file peerj-09-11965-s007.png]
