# Supplementary material for: Comparative transcriptome analysis of R3a and Avr3a-mediated defense responses in transgenic tomato
Source: PeerJ. 2021 Aug 9;9:e11965. doi: 10.7717/peerj.11965 (PMC8359799; doi:10.7717/peerj.11965)
Supplement: Supplemental Information 8 [file peerj-09-11965-s008.docx]

**Table S1. The primers for qRT-PCR**

| **Name** | **Gene** | **Primer sequence (5’—3’)** |
| --- | --- | --- |
| SGT1 | Solyc06g036420.2.1 | F:TCATCTCATCCATGGCGTCC;R:ACGGATCTAACTCAATCGCCT |
| EDS1 | Solyc06g071280.3.1 | F:CGAGTCTTGTTTCTGGGCAG; R:GCTGAGGTGGGAGTGTTTTC |
| RIN4 | Solyc09g059430.3.1 | F:GCTGCAGTTCCAAGATTCGG; R:GGACAACAACACCACTTCCTT |
| ERF68 | Solyc08g078180.1.1 | F:CCGGAGCAACAGCATTGAAT; R:TTTCAACTTTCACCTCCGCC |
| CNGC | Solyc05g050380.3.1 | F:GGTAATCACTCCCAACGCGA; R:GATCTTGTCACACGCATCGC |
| JAZ | Solyc12g009220.2.1 | F:AGGTGACCGGACAGAAATCT; R:AGTTGTGGTGGTAGTAGCTGT |
| NPR1 | Solyc10g079460.1.1 | F:ATCCTAGCCAAACACCTCCC; R:TGGTCCTCGAGCTCAATGTT |
| BAK1 | Solyc10g047140.2.1 | F:GGCCAACTCCAGAAATTGCA; R:CTTCTGAAGCCTGCCCAATG |
| CML | Solyc04g058170.1.1 | F:ATGGTGATGGCCTTGTGTGT; R:GCAACTCTAGCTCCTCTTGGC |
| PR | Solyc07g006710.2.1 | F:GGACTCCAACAGATGCCGTT; R:TGCCCACATAATTGCCCTTCA |
| WRKY | Solyc02g080890.3 | F:CCCAAAATCCAAACGCAGCA; R:TGAAGTTAGGATCGGCGGTG |
| NAC | Solyc06g061080.3.1 | F:TTCTCAGTCGACGGAAGCTG; R:TCCCAAAATCCTACACCGCA |
| MYB | Solyc02g067760.3.1 | F:GGAAAGGGCCTTGGACTATGG; R:TCCGTTCTTCCAGGCAAATG |
| NAC | Solyc03g115850.3.1 | F:AGATTCATCGCCTAGCCACG; R:AATTCCTTAGCGTTGCGGGA |
| ABCG1 | Solyc05g054890.3.1 | F:ACTTTCTACACTTGCGCGGA; R:AATTCCCGGCCCAGAAAGAG |
| ACO2 | Solyc12g005940.2.1 | F:GGTTGCGTGCACATACTGAT; R:CCTCGAGTTGGTCACCTAGG |
| JAR1 | Solyc10g011660.3.1 | F:CCCCTAGATGTGTTGGTCCA; R:TGCTCTATTTGCTCGTTGGC |
| CTR1 | Solyc10g083610.2.1 | F:GTGCAAGAAAGTGGCCGTAT; R:ACCAGTTCCTCTTCTCCAGC |
| PR | Solyc05g054380.2 | F:CAAGGAGATGGTGGAGCTGG; R:CCCAATGGATCCCCTTCAATCA |
| PR | Solyc04g064880.4 | F:ACTGGACAACTTGTTGAGCTCT; R:TCTCCTTTCACAAGTCCTCCA |
| EF1-ɑ | X53043.1 | F:TACTGGTGGTTTTGAAGCTG; R:AACTTCCTTCACGATTTCATCATA |
